# CD8-positive T cells and CD204-positive M2-like macrophages predict postoperative prognosis of very high-risk prostate cancer

**DOI:** 10.1038/s41598-021-01900-4

**Published:** 2021-11-18

**Authors:** Yoshinori Yanai, Takeo Kosaka, Shuji Mikami, Hiroshi Hongo, Yota Yasumizu, Toshikazu Takeda, Kazuhiro Matsumoto, Jun Miyauchi, Shigehisa Kitano, Mototsugu Oya

**Affiliations:** 1 Department of Urology, Keio University School of Medicine, 35 Shinanomachi, Shinjuku-ku, Tokyo, 160-8582 Japan; 2 Department of Diagnostic Pathology, Keio University School of Medicine, Tokyo, Japan; 3 Department of Diagnostic Pathology, Saitama City Hospital, Saitama, Japan; 4 Department of Experimental Therapeutics, National Cancer Center Hospital, Tokyo, Japan; 5 Department of Urology, Keio University School of Medicine, 35 Shinanomachi, Shinjuku-ku, Tokyo, 160-0016 Japan

**Keywords:** Cancer, Immunology, Risk factors, Urology

## Abstract

To stratify the heterogeneity of prostate cancer (PCa) with seminal vesicle invasion (SVI) immunologically after radical prostatectomy focusing on the tumor microenvironment. We retrospectively reviewed the clinicopathological data of 71 PCa patients with SVI, which is known as a factor of very high-risk PCa. Preoperative clinical variables and postoperative pathological variables were evaluated as predictors of biochemical recurrence (BCR) with a multivariate logistic regression. Immune cell infiltration including the CD8-positive cell (CD8^+^ cell) and CD204-positive M2-like macrophage (CD204^+^ cell) was investigated by immunohistochemistry. The cumulative incidence and risk of BCR were assessed with a Kaplan–Meier analysis and competing risks regression. A higher CD8^+^ cell count in the SVI area significantly indicated a favorable prognosis for cancers with SVI (p = 0.004). A lower CD204^+^ cell count in the SVI area also significantly indicated a favorable prognosis for cancers with SVI (p = 0.004). Furthermore, the combination of the CD8^+^ and CD204^+^ cell infiltration ratio of the SVI area to the main tumor area was a significant factor for BCR in the patients with the PCa with SVI (p = 0.001). In PCa patients with SVI, the combination of CD8^+^ and CD204^+^ cell infiltration is useful to predict the prognosis.

## Introduction

Some very high-risk prostate cancer (PCa) patients after radical prostatectomy (RP) experience long-term survival without biochemical recurrence (BCR). BCR is generally defined as the asymptomatic increase of serum prostate-specific antigen (PSA) levels after RP to greater than 0.2 ng/ml^1^. A high Gleason score, positive surgical margins, maximum index tumor diameter, extraprostatic extension, and seminal vesicle invasion (SVI) of PCa were significantly associated with BCR^2–4^. However, some patients with SVI experience long-term survival without BCR after RP. No clinical and pathological characteristics have been accurately stratified in such patients yet.

Although prostate tumor cells are surrounded by a complex tumor microenvironment including host inflammatory or immune effectors, the contribution of the tumor immune response remains unknown^5^. We previously reported that CD204-positive M2-like macrophages (CD204^+^ cell) are associated with prognosis in thymic carcinoma. In that report, CD8- and CD204-positive cells in stroma were identified as possible better prognostic biomarkers, considering the heterogeneity of other biomarkers^6^. The significance of CD204^+^ cell was also reported in patients with lung, kidney, and breast cancer^7^. To better understand the immune profile within prostate tumor local progression, we focused on the two different type of immune cells and the marker localization. One is CD8-positive effector T cell (CD8^+^ cell) as effector cells that play the most important role in the anti-tumor immune response. The other is CD204^+^ cell, which is reported as one of the representative negative prognostic factors^8^. Macrophages can be classified into M1 and M2 subtypes, and CD204 is a marker of M2-like macrophages and plays important roles in the tumor microenvironment by inhibiting anti-tumor immune responses^6,9^. However, there were no study concerning the two different type of immune cells and the marker localization in prostate cancer with SVI. We hypothesized that infiltration patterns of CD8^+^ cell and CD204^+^ cell in the SVI area might differ from those in the main tumor area. The aim of this study was to investigate the clinical, pathological and immunological significance of the tumor immune microenvironment of very high-risk PCa with SVI.

## Materials and methods

### Patients

We retrospectively reviewed the medical records of 1,286 patients who underwent RP at Keio University Hospital and Saitama City hospital from January 2005 to December 2018. The patients’ serum PSA levels were assessed after RP. A postoperative PSA level greater than 0.2 ng/ml was defined as BCR. Inclusion criteria was SVI and we excluded patients who were lacking data. 7 patients who received adjuvant therapy and 8 patients without a nadir PSA level less than 0.2 ng/ml were also excluded. Finally, we analyzed 71 (5.5%) patients. This study was approved by the Institutional Review Board of Keio University and Saitama City Hospital. Informed written consent was obtained from the study participants.

### Pathological analysis

The Gleason scores after the RP were cited from pathological reports. In patients who received neoadjuvant androgen deprivation therapy, Gleason scores obtained from a needle biopsy were used instead of prostatectomy Gleason scores. All PCa cases were histologically diagnosed before RP by ultrasound-guided needle biopsy via the transrectal or transperineal approach. After RP, all specimens were fixed in 10% formalin and embedded in paraffin. Whole-mount section pathology was performed, and all specimens were cut into thin slices perpendicular to the urethra from the apex to the base after removing the seminal vesicle. All seminal vesicles were cut longitudinally. All slides were stained with hematoxylin and eosin. The Gleason score of the PCa and the other pathological parameters, including SVI, were evaluated in each section. SVI was defined as cancer invasion into the extraprostatic portion of the seminal vesicles.

### Immunohistochemistry

Immunohistochemistry was performed after the sections were formalin-fixed and paraffin-embedded. The sections were deparaffinized in xylene and then rehydrated in graded alcohols and distilled water. After antigen retrieval with citric acid (pH 6.0), endogenous peroxidase activity was blocked with 1% hydrogen peroxide for 30 min, followed by washing with distilled water. To bind nonspecific antigens, the sections were incubated with 5% skim milk for 15 min. The sections were incubated with an anti-CD8 rabbit polyclonal antibodies (1:100 dilation, Abcam, Cambridge, MA, USA) and anti-CD204 rabbit polyclonal antibodies (1:100 dilation, Abcam, Cambridge, MA, USA) at room temperature for one hour, followed by conjugation to the secondary antibody and DAB staining.

### Evaluation of immunostaining

To evaluate CD8 and CD204 staining, the cells with positive staining were counted in five representative fields (200 μm^2^/field) using light microscopy. The median number of the CD8^+^ and CD204^+^ cell was estimated for each main tumor area and SVI area. Values above median number of the CD8^+^ or CD204^+^ cells were considered as CD8-high or CD204-high. Values under median number of the CD8^+^ or CD204^+^ cells were considered as CD8-low or CD204-low. The experienced urologic pathologists, blinded to the patients’ clinical data, performed the counting.

### SVM (the Seminal Vesicle area to the Main area) Score

Focusing on the distribution of the CD8^+^ and CD204^+^ cells in each tumor slide, we calculated the ratio of the CD8^+^ and CD204^+^ cell counts in the SVI area to those in the main tumor area for each patient. We defined the ratio as “*SVM (the Seminal Vesicle area to the Main area) Score*”. We calculated estimates (with confidence intervals) of the best-fit receiver operating characteristic (ROC) curve and the corresponding area under the ROC curve, and then calculated the cutoff value of each *SVM Score* for CD8^+^ and CD204^+^ cells, respectively.

#### Statistical analysis

Differences in continuous variables between groups were evaluated using the Mann–Whitney U test. The Chi-squared test was used to analyze the difference in the number of patients between two groups. To identify factors predictive of BCR, univariate and multivariate analyses were performed using the Cox proportional hazards model with stepwise forward selection. Kaplan–Meier curves were drawn to evaluate postoperative BCR-free survival. All reported p-values were two-sided, and statistical significance was set at 0.05. The statistical analyses were performed using the R Statistical Language version 3.5.3 program (https://www.r-project.org) and the SPSS version 25.0 statistical software package (https://www.ibm.com/analytics/spss-statistics-software).

#### Ethics approval and consent to participate

Written informed consent was obtained from all patients included in the study. This study was approved by the Institutional Review Board of Keio University and Saitama City Hospital and was performed in accordance with the Declaration of Helsinki.

## Results

### Patients characteristics of seminal vesicle invasion

The clinicopathological data of the 71 patients with SVI are summarized in Table 1. The median PSA value at biopsy was 9.1 ± 8.6 ng/ml. The median prostate volume at biopsy was 30.0 ± 12.2 ml. The median PSA density at biopsy was 0.36 ± 0.51 ng/ml/ml. SVI was not detected (clinical T3b) in any patients before RP.

**Table.1:** Patient's characteristics.

	N	Median ± SD (5–95% CI)
**Age at operation (yr)**		68.4 ± 5.7 (55.5–75.3)
<66	24 (33.8%)	
≧66	47 (66.2%)	
**PSA value at biopsy (ng/ml)**		9.1 ± 8.6 (4.7–32.5)
<10	39 (54.9%)	
10–20	23 (32.4%)	
>20	8 (11.3%)	
Unknown	1 (1.4%)	
**Prostate volume at biopsy (ml)**		30.0 ± 12.2 (13.7–51.9)
<30	34 (47.9%)	
≧30	34 (47.9%)	
Unknown	3 (4.2%)	
**PSA-density at biopsy (ng/ml/ml)**		0.36 ± 0.51 (0.12–1.18)
<0.20	15 (21.1%)	
≧0.20	53 (74.6%)	
Unknown	3 (4.2%)	
**Clinical T stage**		
cT1c, 2a, 2b	47 (66.2%)	
cT2c, 3a	22 (31.0%)	
Unknown	2 (2.8%)	
**Grade group**		
1, 2	14 (19.7%)	
3, 4, 5	57 (80.3%)	

### Immune cell infiltration in prostate tissue

**Figure 1:**
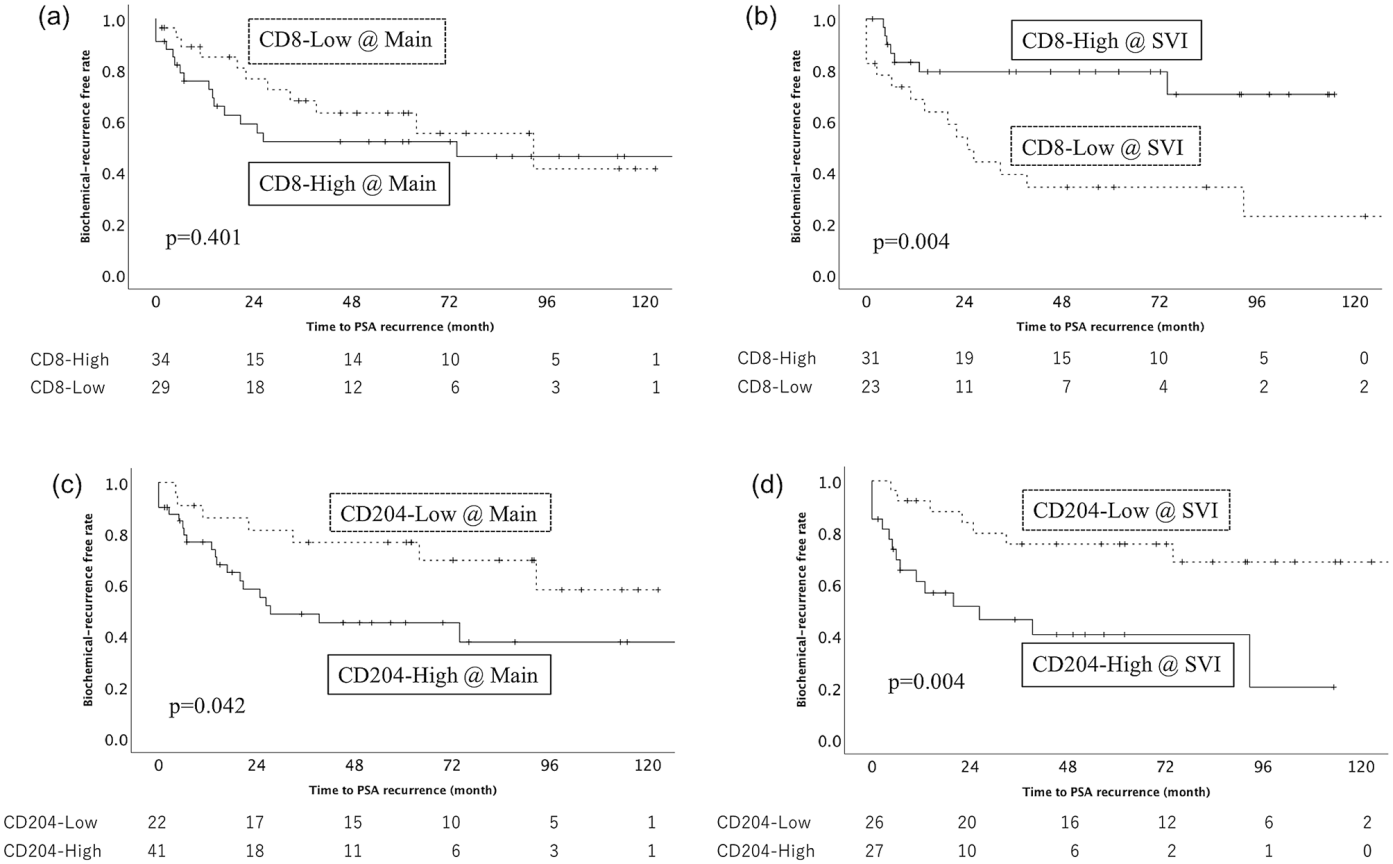
The Kaplan–Meier analysis of the recurrence-free survival of patients with seminal vesicle invasion according to the CD8^+^ cell in the main tumor area (a, p = 0.401) and seminal vesicle invasion area (b, p = 0.004) and the CD204^+^ cell in the main tumor area (c, p = 0.042) and seminal vesicle invasion area (d, p = 0.004). Solid square means higher density, and dotted square means lower density.

The median CD8^+^ cell count in the main tumor area was 40.0 ± 75.4 (95% CI: 5.0–189.0) cells/mm^2^ compared with 30.0 ± 42.7 (95% CI: 3.75–150.0) cells/mm^2^ in the SVI area. No significant difference was observed in BCR between the higher and lower CD8^+^ cell count in the main tumor area (p = 0.401, Fig. 1a). However, the statistically significant difference was observed in BCR between the higher and lower CD8^+^ cell count in the SVI area, which suggested that a higher CD8^+^ cell count in the main tumor area predicted a favorable prognosis for PCa with SVI (p = 0.004, Fig. 1b).

The median CD204^+^ cell count in the main tumor area and the SVI area was 300.0 ± 139.9 (95% CI: 50.0–540.0) cells/mm2 and 300.0 ± 163.1 (95% CI: 25.0–600.0) cells/mm^2^, respectively. The statistically significant difference was observed in BCR between the higher and lower CD204^+^ cell count in the main tumor area, which suggested that a higher CD204^+^ cell count in the main tumor area predicted a worse prognosis for PCa with SVI (p = 0.042, Fig. 1c). In the SVI area, the statistically significant difference was also observed in BCR between the higher and lower CD204^+^ cell count (p = 0.004, Fig. 1d).

### Combination of immune cell infiltration

**Figure 2:**
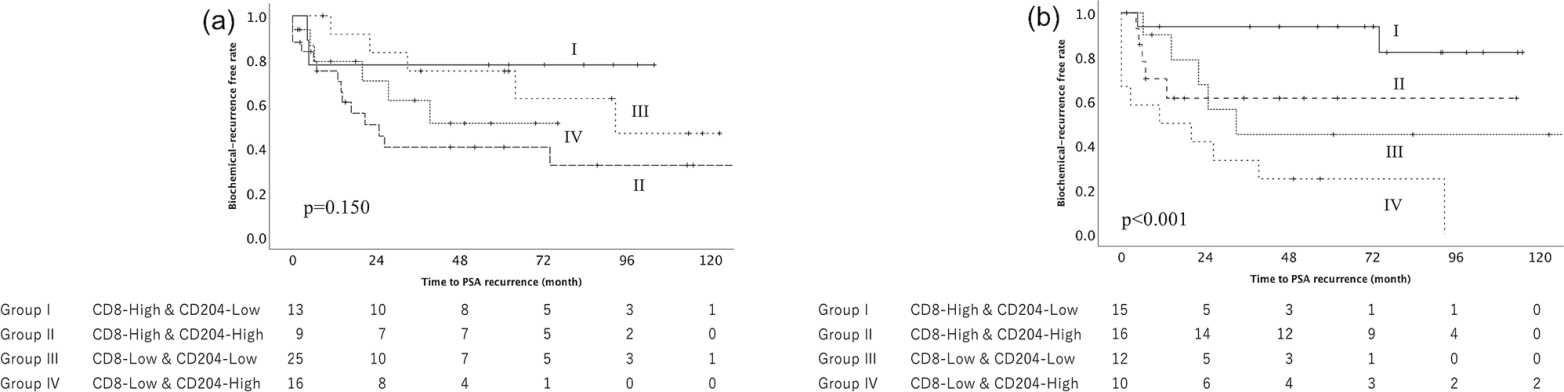
The Kaplan–Meier analysis of the recurrence-free survival of patients with seminal vesicle invasion according to the combination of the CD8^+^ and CD204^+^ cell. No significant difference between four groups was observed in the main tumor area (a, p = 0.150). However, a significant difference between four groups was observed in the seminal vesicle invasion area (b, p < 0.001). Group II, which included patients with the higher CD8^+^ cell count and the lower CD204^+^ cell count, had the most favorable outcome among the four groups. The 6-year progression free survival (PFS) rate of Group II was 90.9%.

The combination of the CD8^+^ and CD204^+^ cells in the main tumor area and SVI area was evaluated in each patients. Each patients were classified into four groups according to the amount of the CD8^+^ and CD204^+^ cells. Group I included patients with a higher CD8^+^ cell count and a lower CD204^+^ cell count. Group II included patients with a higher CD8^+^ cell count and a higher CD204^+^ cell count. Group III included patients with a lower CD8^+^ cell count and a lower CD204^+^ cell count. Group IV included patients with a lower CD8^+^ cell count and a higher CD204^+^ cell count. No significant difference among four groups was observed in the main tumor area (p = 0.150, Fig. 2a). However, a significant difference between four groups was observed in the SVI area (p < 0.001, Fig. 2b). Group I had the most favorable outcome among the four groups. The 5-year progression free survival (PFS) rate of Group I was 90.9%. Group II and Group III had the intermediate outcome. The 5-year PFS rate of Group II and III was 66.7% and 50.0%, respectively. Group IV had the worst outcome. The 5-year PFS rate of Group IV was 25.0%.

### Logistic regression analysis

Univariate Cox regression revealed the serum PSA level, the CD8^+^ cell count in the SVI area and the CD204^+^ cell count in the SVI area had significant impact on BCR (p = 0.001, p = 0.002 and p = 0.036, respectively, Table 2). Among the clinicopathologic parameters analyzed, multivariate Cox regression revealed that the higher serum PSA level (Hazard Ratio (HR) = 2.870, p = 0.004), the lower CD8^+^ cell count (HR = 3.004, p = 0.018) and the higher CD204^+^ cell count in the SVI area (HR = 3.290, p = 0.013) are an indication of the unfavorable prognosis of cancers with SVI (Table 2).

**Table.2:** Biochemical recurrence free survival according to Cox proportional hazards analysis.

Variable	Univariate	Multivariate			
		Hazard ratio	5% CI	95% CI	p-value
Age at operation (yr)	0.115				
PSA value at biopsy (ng/ml)	0.001	2.870	1.399	5.889	0.004
Grade Group	0.371				
CD8^+^ cell count in the main tumor area	0.469				
CD8^+^ cell count in the seminal vesicle invasion area	0.002	3.004	1.207	7.478	0.018
CD204^+^ cell count in the main tumor area	0.198				
CD204^+^ cell count in the seminal vesicle invasion area	0.036	3.290	1.282	8.443	0.013

## SVM (the Seminal Vesicle area to the Main area) Score

**Figure 3:**
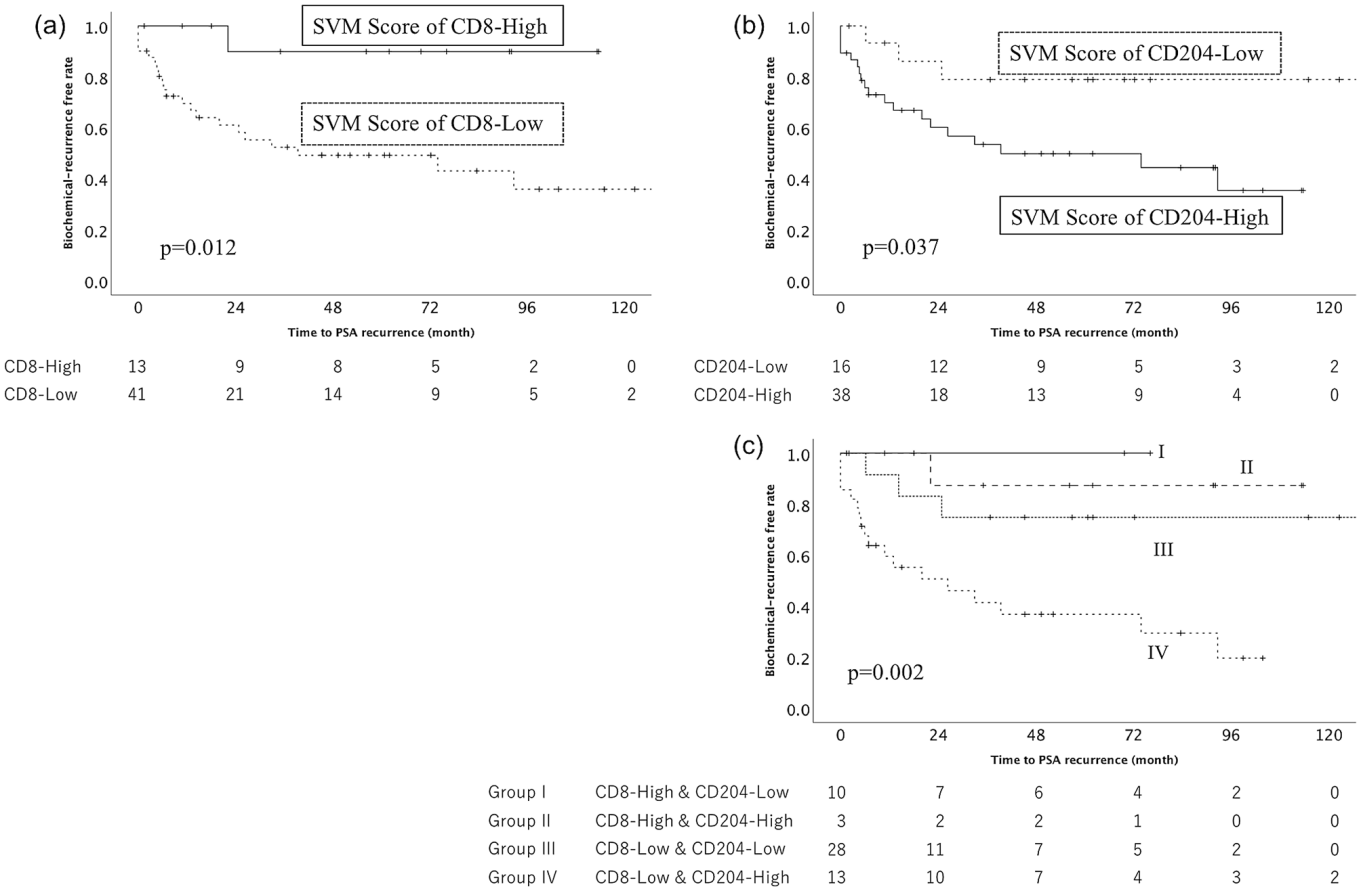
The Kaplan–Meier analysis of the recurrence-free survival of patients with seminal vesicle invasion according to the ratio of the CD8^+^ and CD204^+^ cells in the seminal vesicle invasion area to those in the main tumor area (*SVM Score*) for each tumor. A significant difference between the higher and lower *SVM Score* of CD8 + and CD204^+^ cell count was observed (CD8: p = 0.012, 4a, CD204: p = 0.037, 4b). Group I, which included patients with the higher *SVM Score* of CD8^+^ cell count and the lower *SVM Score* of CD204^+^ cell count, had the significantly favorable outcome (p = 0.002, 4c).

Each cut-off value of *SVM Score* of CD8^+^ and CD204^+^ cell was 1.5 and 0.6, respectively. A statistically significant difference in the *SVM Score* of the CD8^+^ cell was observed (p = 0.012, Fig. 3a). It suggested that the higher *SVM Score* of the CD8^+^ cell predicted a favorable prognosis of PCa with SVI. A statistically significant difference in the *SVM Score* of the CD204^+^ cell was also observed (p = 0.037, Fig. 3b). It suggested that the lower *SVM Score* of the CD204^+^ cell predicted a favorable prognosis of PCa with SVI. A statistically significant difference in the combination of the *SVM Score* of the CD8^+^ and CD204^+^ cell was observed among the four groups (p = 0.002, Fig. 3c).

## Discussion

SVI is an independent prognostic factor of PCa, and in general, adjuvant radiotherapy is effective in patients with SVI^10^. However, this study revealed that the higher CD8^+^ cell count and the lower CD204^+^ cell count in the SVI area are an indication of the favorable prognosis of cancers with SVI.

Several studies have been reported that CD8^+^ cell infiltration is associated with better prognosis^11,12^. In other reports, CD8^+^ cell was also associated with recurrence, progression, or lymph node invasion^13,14^. CD8^+^ cell may be associated with cancer progression by inhibiting the activity of effector T cells^15,16^. Thus, we had hypothesized that higher infiltration of CD8^+^ cell could be a favorable prognostic factor of BCR, however, no significant difference was observed in BCR between the higher and lower CD8^+^ cell count in the main tumor area (Fig. 1a). Then, we focused on the distribution of the CD8^+^ cell in each tumor slide. CD8^+^ cell secret cytotoxic molecules and cytokines, including perforin, granzyme, interferon-gamma, tumor necrosis factor-alpha and so on, which elicit anti-tumor effects. They are released only in the direction of the target cell by identifying cancer antigen-derived epitopes presented on MHC molecules on tumor cells by T cell receptors to avoid non-specific bystander damage to normal tissue^17^. The mechanism might suggest our data that CD8^+^ cell increased in the SVI area rather than the main tumor area in each unfavorable tumors was correct (Fig. 1b).

On the other hand, tumor-associated macrophage (TAM) infiltration in the tumor microenvironment is directly associated with tumor invasion, nodal status, and clinical stage in some cancers, and TAMs also have been reported to regulate the growth of prostate cancer^18–20^. A previous study reported that M2 TAMs inhibit cytotoxic CD8^+^ cell, resulting in weakened anti-tumor immunity and increased tumor-infiltrating CD8^+^ cells^21^. We also focused on the distribution of CD204^+^ cell in each tumor slide as well as CD8^+^ cell. CD204 is not M2 specific, and CD163 might be suitable for M2-like marker^22^. In recent article, high number of CD163-positive cells was related to poor clinical course in prostate cancer, however, M1 cells were also associated to poor clinical course^23^. In breast cancer, the data that cancer-derived factor induced CD204 expression in cultured macrophages rather than CD163 and CD204^+^ cells predicted poor clinical course rather than CD163 indicated that CD204 might be a marker for protumor phenotype more suitable than CD163 in some organs^24^. Macrophages express PD-L1 and PD-L2, and macrophages in SVI can be considered positive for PD-L1 and L2, which might be related to immune suppression. We will evaluate PD-L1 and L2 in our future study. In our present study, a significant difference between the higher and lower CD204^+^ cell count in the main tumor area and the SVI area was observed in BCR (Fig. 1c and 1d).

Patients with very high-risk PCa, including SVI are usually considered to be candidates for adjuvant therapy^25–27^. However, it is uncertain whether all patients need adjuvant therapy, as some of them do not appear to benefit. Therefore, the development of an improved prediction model of the very high-risk PCa is necessary and unmet needs. In this study, we assessed the relationship of the distribution of the CD8^+^ and CD204^+^ cell. The combination of those different immune cells could be more useful for predicting the prognosis of the very high-risk PCa than a single immune cell. This study was derived from a retrospective review of patients treated at two institutions and the specimens used in this study could not reflect all status of the tumor immune microenvironment. The small tumor volume in SVI is also difficult to evaluate. However, the findings obtained in this study could be useful for clinical assessment and decision-making for PCa patients with SVI after RP. It is highly worthwhile to verify these results focused on the different type of immune cells and the marker localization in future prospective studies.

In conclusion, in prostate cancer patients with SVI, the combination of the CD8^+^ and CD204^+^ cell infiltration in the SVI area is useful to predict the prognosis.

## Supplementary Information

Supplementary Information 1.

Supplementary Information 2.
