# Supplementary figures and images for: CD8-positive T cells and CD204-positive M2-like macrophages predict postoperative prognosis of very high-risk prostate cancer
Source: Sci Rep. 2021 Nov 18;11:22495. doi: 10.1038/s41598-021-01900-4 (PMC8602636; doi:10.1038/s41598-021-01900-4)

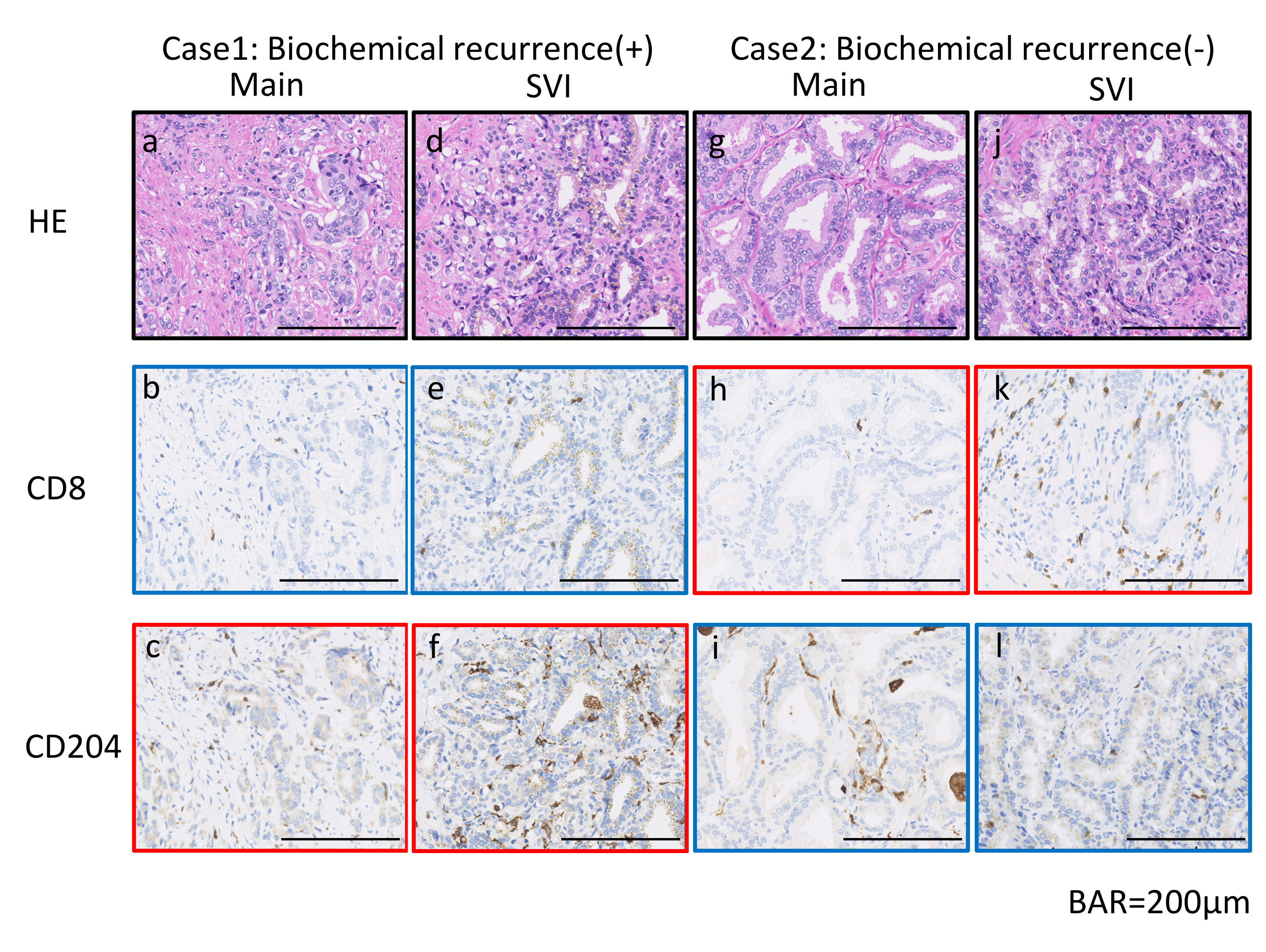

Supplement: Supplementary file 1 — Supplementary Information 1. [file 41598_2021_1900_MOESM1_ESM.png]
