# Supplementary material for: CD8-positive T cells and CD204-positive M2-like macrophages predict postoperative prognosis of very high-risk prostate cancer
Source: Sci Rep. 2021 Nov 18;11:22495. doi: 10.1038/s41598-021-01900-4 (PMC8602636; doi:10.1038/s41598-021-01900-4)
Supplement: Supplementary file 2 — Supplementary Information 2. [file 41598_2021_1900_MOESM2_ESM.docx]

**Supplemental Figure.**

The representative immunohistochemical staining of the CD8^+^ and CD204^+^ cell infiltration in the main tumor area and seminal vesicle invasion area. Biochemical recurrence occurred in Case 1 (a-f). The lower CD8^+^ cell density was detected in the seminal vesicle invasion area (e) than main tumor area (b). The higher CD204^+^ cell density was detected in the seminal vesicle invasion area (f) than main tumor area (c). Biochemical recurrence did not occur in Case 2 (g-l). The higher CD8^+^ cell density was detected in the seminal vesicle invasion area (k) than main tumor area (h). The lower CD204^+^ cell density was detected in the seminal vesicle invasion area (l) than main tumor area (i). Red square means higher density, and blue square means lower density.
